# Non-parametric Algorithm to Isolate Chunks in Response Sequences

**DOI:** 10.3389/fnbeh.2016.00177

**Published:** 2016-09-21

**Authors:** Andrea Alamia, Oleg Solopchuk, Etienne Olivier, Alexandre Zenon

**Affiliations:** Institute of Neuroscience, Université catholique de LouvainBruxelles, Belgique

**Keywords:** chunking, sequence learning, working memory, segmentation, concatenation

## Abstract

Chunking consists in grouping items of a sequence into small clusters, named chunks, with the assumed goal of lessening working memory load. Despite extensive research, the current methods used to detect chunks, and to identify different chunking strategies, remain discordant and difficult to implement. Here, we propose a simple and reliable method to identify chunks in a sequence and to determine their stability across blocks. This algorithm is based on a ranking method and its major novelty is that it provides concomitantly both the features of individual chunk in a given sequence, and an overall index that quantifies the chunking pattern consistency across sequences. The analysis of simulated data confirmed the validity of our method in different conditions of noise, chunk lengths and chunk numbers; moreover, we found that this algorithm was particularly efficient in the noise range observed in real data, provided that at least 4 sequence repetitions were included in each experimental block. Furthermore, we applied this algorithm to actual reaction time series gathered from 3 published experiments and were able to confirm the findings obtained in the original reports. In conclusion, this novel algorithm is easy to implement, is robust to outliers and provides concurrent and reliable estimation of chunk position and chunking dynamics, making it useful to study both sequence-specific and general chunking effects. The algorithm is available at: https://github.com/artipago/Non-parametric-algorithm-to-isolate-chunks-in-response-sequences.

## Introduction

Chunking refers to a strategy supposedly used to deal with the limitation of working memory capacity (Miller, 1956; Ericsson et al., 1980; Gobet et al., 2001), which consists in grouping sequence items together (Cowan, 2001, 2010), on the basis of their temporal contingency, feature similarity, or spatial vicinity (Terrace, 1991; Verwey, 1996; Gobet and Simon, 1998; Gobet et al., 2001; Sakai et al., 2003; Miyapuram et al., 2006; Bor and Seth, 2012; Mathy and Feldman, 2012; Wymbs et al., 2012). Chunking has already been the subject of many studies, in particular in the context of sequence learning (Sakai et al., 2003; Boyd et al., 2009; Perlman et al., 2010; Tremblay et al., 2010). It is usually regarded as a mechanism that allows us to process sequences more efficiently (Sakai et al., 2003; Perlman et al., 2010), although some studies have failed to find a correlation between chunking and sequence learning or sequence performance (Clerget et al., 2012; Wymbs et al., 2012). The formation of chunks has been seen as the result of two distinct processes occurring sequentially (Wymbs et al., 2012). The first one is known as “segmentation” (Sakai et al., 2003; Bo and Seidler, 2009), and consists in breaking the sequence into numerous small chunks. The second process, known as “concatenation,” is thought to occur at a later stage during learning; it consists in assembling several short chunks into longer segments (Verwey, 1996; Wymbs et al., 2012), allowing to store a larger number of items at once.

Chunks are recognizable because they lead to a typical pattern in reaction time (RT) series. Indeed, the retrieval of a chunk comes at the cost of longer RT at chunk onset, suggesting that all the elements of the chunks are retrieved prior to execution of the first chunk element (Verwey, 1996; Clerget et al., 2012). However, identifying chunks on the basis of this feature remains a challenge because of the high variability of RT and because of the dynamic nature of chunks, which fluctuate during training, across block repetitions (Wymbs et al., 2012). Most of the methods described so far in the literature provide only global indexes that quantify how much of the RT variability can be explained by the chunking patterns, but without reporting the number or position of chunks within the sequence (see Clerget et al., 2012 for an interesting exception, but with some methodological limitations). Therefore, a statistically valid and unbiased method allowing not only to track the overall chunking dynamics but also to explore how chunking strategies affect performance in sequence processing is currently lacking. Here we propose a novel algorithm that naturally combines the capacity to detect chunk positions and to quantify overall chunk consistency, in response sequences including at least 4 repetitions per block. This algorithm was first validated on simulated data, and then on 3 datasets previously acquired in the laboratory (Clerget et al., 2012; Alamia et al., 2016; Solopchuk et al., 2016).

## Methods

### The ranking algorithm

**Figure 1:**
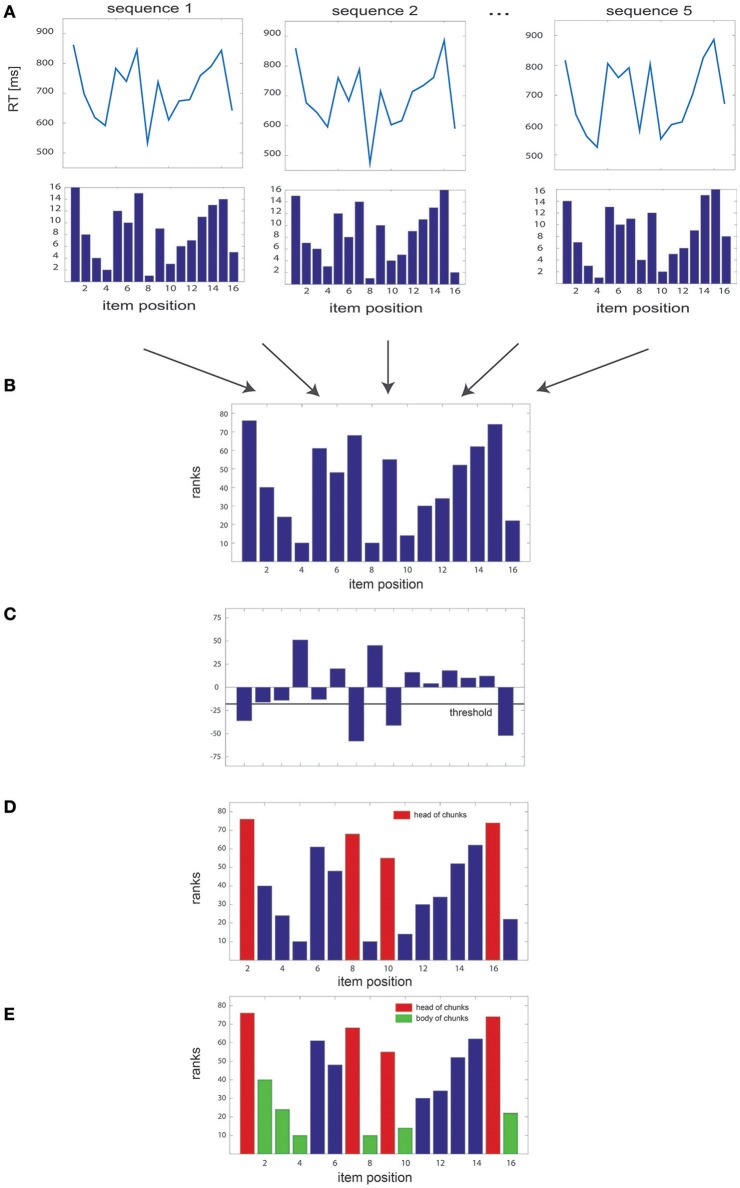
**Overview of the algorithm steps. (A)** Simulated RT series, with the corresponding rank of each of the 16 items of the sequence, illustrated for several sequence repetitions. **(B)** Sum of the ranks gathered for all sequences of the block. **(C)** Pairwise differences between rank sums, with the threshold used to identify the heads of the chunks. **(D)** Sum of the ranks of each item in the sequence, with identified head of chunks highlighted in red; **(E)** same as in **(D)** with the body of chunks highlighted in green.

We implemented a non-parametric algorithm designed to disclose chunks by uncovering specific patterns in RT series. This algorithm is based on a ranking method that assigns a score to each item of a sequence of length N and repeated k times in a block; this score is subsequently used to detect chunks at the block level. Overall, the algorithm could be summarized in five steps (see Figure 1):

1. For each sequence of length N, the RTs of each sequence items are ranked in ascending order, such that a value of 1 is assigned to the item associated with the shortest RT and a value of N to the item associated with the largest one (Figure 1A). Missing values in the dataset, e.g., due to time-out constrains or lack of response, are replaced with the average RT obtained from the same items in the remaining sequences of the block. In the very unlikely case in which the same RT was obtained in several items, the algorithm ranks them in order of appearance (i.e., highest rank to the first item).
2. Then, we sum the rank values of each item gathered for the k sequences of the block, leading to a total of N summed rank values (Figure 1B).
3. The difference between the summed rank values of each pair of consecutive items is then computed: a negative value indicates that the first item of the pair has a higher rank and, therefore, an overall larger RT than the second one; a positive value reveals the opposite relationship between RTs (Figure 1C).
4. Pairwise rank differences falling below a given threshold θ (i.e., overall larger RTs, see below for the details of threshold computation) indicate heads of chunks (Figure 1D).
5. Finally, when one item is identified as a chunk head, the subsequent items in the sequence are considered as belonging to the same chunk (chunk “body”) provided the 2 following conditions are fulfilled: 1) the difference between the rank of the chunk head and that of each subsequent item of the sequence is below the threshold θ and 2) the absolute difference in ranks between all pairs of successive items (excluding the head) is smaller than the median of the absolute rank differences in the block (Figure 1E). This second criterion was included so as to ensure that all items within a chunk had comparable ranks.

The θ threshold is estimated by computing, for each block, the theoretical distribution of the pairwise rank differences. The threshold is estimated for each set of time series (see Figure S1A) depending on the number of items in the sequence (*n*), and the number of sequences in a block (*k*). Given that the probability of getting a given rank is the same for all sequence items, the probability mass function of item ranks follows a uniform distribution:

(1) $$\begin{array}{l} F\left(x\right)=U\;\left\{1,n\right\} \end{array}$$

The probability distribution of the summed rank values was obtained by convolving the uniform distribution k times (Equations 2, 3).

(2) $$\begin{array}{l} F^{\text{1}}\left(x\right)=U\;\left\{1,n\right\}*U\;\left\{1,n\right\} \end{array}$$

(3) $$\begin{array}{l} F^{k}\left(x\right)=F^{k-1}\left(x\right)*U\;\left\{1,n\right\} \end{array}$$

Then, to determine the distribution of the difference of successive sums of ranks, we convolved the obtained distribution *F* ^*k*^*(x)* with itself and centered it on zero (Equations 4, 5).

(4) $$\begin{array}{l} G\;\left(x\right)=F^{k}\left(x\right)*F^{k}\left(x\right) \end{array}$$

(5) $$\begin{array}{l} D\;\left(x-E\left[G\right]\right)=G\;\left(x\right) \end{array}$$

Finally, the theoretical distribution of the difference of rank sums, and therefore the threshold for determining the significance level given a type-I error rate α, is as follows:

(6) $$\begin{array}{l} \theta =max\;\left(x\right):\underset{xi\leq x}{\sum }D\;\left(xi\right)\leq \;\alpha \end{array}$$

A Matlab script performing these computations is provided at: https://github.com/artipago/Non-parametric-algorithm-to-isolate-chunks-in-response-sequences.

However, in order to simplify the procedure, we also devised a rule of thumb that can be used to compute the threshold, given N and k. We fitted the thresholds obtained with the theoretical approach described above, for a range of k (from 2 to 20) and N values (from 2 to 64) and for an α *of 0.05*. This led to the following formula:

(7) $$\begin{array}{l} \theta =-0.5-0.70647*\surd \left(N\right)*k \end{array}$$

This fitting procedure is also provided as a Matlab script in https://github.com/artipago/Non-parametric-algorithm-to-isolate-chunks-in-response-sequences.

This algorithm is quite robust to outliers, since it is non-parametric and considers the data block-wise, thus reducing the influence of outliers occurring randomly in one sequence. The algorithm is also robust, in principle, to type I error, avoiding the identification of false chunks: the threshold, set by default to α = 0.05, could be tuned to increase or reduce the sensitivity of the algorithm in detecting chunks, depending on the desired degree of accuracy and false discovery rate.

Furthermore, a chi-square test was performed on the summed rank values computed at the block-level (Figure 1B) to assess how much these differed from a uniform distribution. The obtained chi-square index (referenced later as the chi^2^-index) provides us with a quantitative evaluation of the chunking pattern for each block, and can be interpreted as an index of the chunking consistency: the bigger the distance from the uniform distribution, the more consistent the chunking pattern within the blocks.

### Simulated datasets

To test the validity of our algorithm, we generated 5 different structured datasets composed of sequences of 16 items (*N* = 16) repeated 5 times in each block (*k* = 5). The different chunking patterns were obtained by embedding in the sequence either 2 (few) or 4 (many) chunks composed of either 2 (short) or 4 (long) items each. There was no chunking pattern in the fifth condition, which was used as a control condition. The number of sequences per block played an important role in the efficacy of the algorithm (see the Results section for details). Indeed, since chunks are detected block by block, the higher the number of sequence repetitions, the smaller the influence of outliers. In contrast, the length of the sequence had little influence on the outcome of the algorithm, and the value of 16 was chosen to fit the experimental datasets (see below).

Specifically, each sequence was built according to the following formula:

1. rt = rt_0_ + noise.
2. noise = (rt_0_ * slope + intercept) * N(0,1).

where rt_0_ represents the baseline level of the RT, and could take three possible values:

- 760 ms for the non-chunk items,
- 810 ms for the heads of chunks,
- 495 ms for the body of chunks.

Those values were estimated by averaging, from the actual dataset #1 (see below), the RT of a naïve (non-chunk items) and a non-naïve participant (head and body of chunks) performing a known hierarchically structured sequence (Solopchuk et al., 2016). For all simulated datasets, the chunks were distributed randomly in the sequences, avoiding overlaps between chunks, or pieces of chunks at the end of the sequence. The chunk positions were kept constant in all the sequences of each block.

The noise added in the simulated data (see formula 3) was normally distributed. This choice of noise distribution is conservative since it leads to larger overlaps between the RT distributions of the different chunk categories than would, for instance, a log-normal distribution of equal variance and mean. In the experimental dataset #1 (see below), we found a strong correlation for each subject between the standard deviation (SD) and mean (μ) of each block. To account for this fact, we estimated the coefficients of the linear regression between SD and μ and we applied a similar linear relation between SD and μ in our simulated dataset, as shown in the formula. Therefore, the range of *slope* and *intercept* parameters included in the simulated dataset were estimated from dataset #1, because it comprises the largest sample and did not involve experimental manipulation (i.e., TMS). The slope values in this dataset ranged between −0.1 and 0.8, and the intercept from −12 to 12. We sampled these ranges of values such that we included 9 bins for the intercept (steps of 0.1) and 24 bins for the slope (steps of 1), yielding a 9 × 24 (slopes × intercept) matrix. The *slope* and *intercept* parameters estimated from datasets #2 and #3 provided similar values as those of dataset #1. Moreover, in the experimental data, high intercepts were associated with low slope values, and large slopes to low intercept values; therefore, low values of both slope and intercept (i.e., very small noise level) or high values of both slope and intercept (i.e., very high noise level) are theoretical conditions useful to evaluate the algorithm, but exceptional in experimental datasets.

### Experimental datasets

As already mentioned, we also tested our algorithm on three “real” datasets (Clerget et al., 2012; Alamia et al., 2016; Solopchuk et al., 2016).

Dataset #1 was gathered from 26 participants (16 women, mean age = 27 years, *SD* = 4) who were asked to learn explicitly a 16-digit sequence. Participants had to choose between two digits displayed simultaneously on a computer screen by pressing on the left or right mouse button; one digit was the “target” i.e., the digit belonging to the sequence, the other one was a distractor (Kühn, 2011). The position (left or right) of the “target” was pseudo-randomized, ensuring that the sequence of digits was not associated with any systematic sequence of motor responses. The experiment comprised 8 blocks, each of which being composed of 6 sequence repetitions. The sequence, organized in distinct hierarchical levels, was 3232232341411414 (see Figure 1; Alamia et al., 2016). Moreover, before the task, all the subjects underwent a set of working memory tasks for around 30 min (Solopchuk et al., 2016).

The dataset #2 was gathered from 24 participants (12 women, mean age = 23 years *SD* = 4) who performed the same task as aforementioned, but with each block being composed of 5 sequence repetitions. The participants received a continuous theta-burst TMS (cTBS) before the experiment either over the vertex (control group) or over Broca's area in order to determine the role of this cortical area in high-level chunking (Alamia et al., 2016).

Dataset #3 was gathered in an experiment performed on 17 participants (9 women, mean age = 27, *SD* = 4), who performed 8 blocks of a Serial Reaction Time Task (SRTT), in which a sequence of 20 items was learnt implicitly. The sequence was 31422413424131321234, in which the numbers correspond to fixed finger-response key mappings, from index (1) to pinkie (4). In block 7 the sequence was shuffled, in order to test the sequence-specific learning (Clerget et al., 2012). All the participants received cTBS before learning the sequence: in one group, cTBS was delivered over the caudal part of the Broca's area (left BA44), while in the other group, it was applied, as a control, over the vertex (Clerget et al., 2012).

### Analysis

We first evaluated the performance of our algorithm on the simulated datasets, and then on the 3 experimental datasets.

#### Simulated dataset

A Monte Carlo approach (1000 iterations) was performed in order to test our algorithm in a supervised way, as the number of chunks, their position and length were known. Specifically, we simulated different RT series for each condition (i.e., no chunks, two 2-item chunks, four 2-item chunks, two 4-item chunks, and four 4-item long chunks) and for different values of noise, i.e., *intercept* and *slope* (see above). The outcome measures were the number of chunks identified by our algorithm, their length and their position in the sequence. For each of these outcomes, we tested whether the estimated confidence intervals included the known chunk parameters. Furthermore, we computed the confidence intervals of the chi^2^-values obtained in each condition for each combination of noise parameters, and determined whether they overlapped with those obtained with the random dataset (i.e., the dataset that included no structure in the RT sequences).

In order to analyze how the results of the algorithm varied according to the number of sequences per block (k), we performed the same Monte Carlo analysis (*n* = 1000) while varying k (2, 4, 5, 6, 8, or 10), and we assessed the number of chunks identified, their length and the error of the identified position by means of confidence intervals. The aim of this analysis was to test the reliability of the algorithm for different sequence numbers. It was performed considering only one point in the slope/intercept space, which was the mean value of all the subjects from the experimental dataset #1.

#### Experimental datasets

In the second series of analyses, we tested the algorithm on three datasets gathered during different experimental sessions described above. For each dataset we compared the results obtained with our algorithm and those obtained in the original studies. Moreover, for each dataset we ran three GLMM (Generalized Linear Mixed Model), considering as dependent variable either the number of the chunks, the length of the chunks, or the chi^2^-index. The BLOCK number factor was included as a fixed effect. When appropriate, we included the factor GROUP to discern those who received TMS pulses over BA44 or the vertex (i.e., dataset #2 and #3). The random model was implemented considering all the factors included in the fixed model. In case of a lack of significant effect of the factor BLOCK, the Bayes Factor was computed by comparing the models with and without the BLOCK factor. We also performed Spearman correlations between the chi^2^-index and response accuracy or RT, in order to unveil a possible effect of chunking on performance.

All the analyses regarding the GLMM were performed in SAS 9.3 (SAS Institute, Cary NC), whereas the other analyses were performed in Matlab 7.5 (The MathWorks, Natick, Massachusetts, USA).

## Results

### Simulated datasets

**Figure 2:**
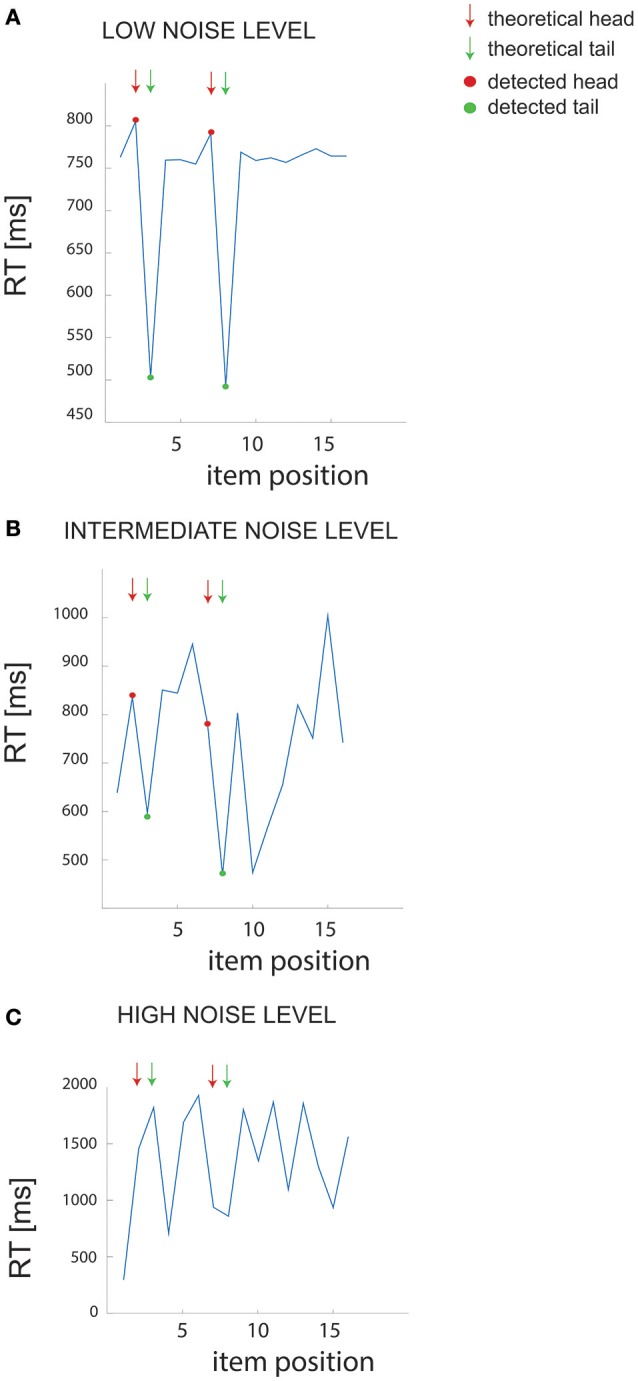
**Example of RT data. (A–C)** Example of RT data for different noise levels in the simulated dataset (few and short chunks). The arrows point to the “theoretical” chunks, while the red and green dots show the chunks detected by our algorithm.

**Figure 3:**
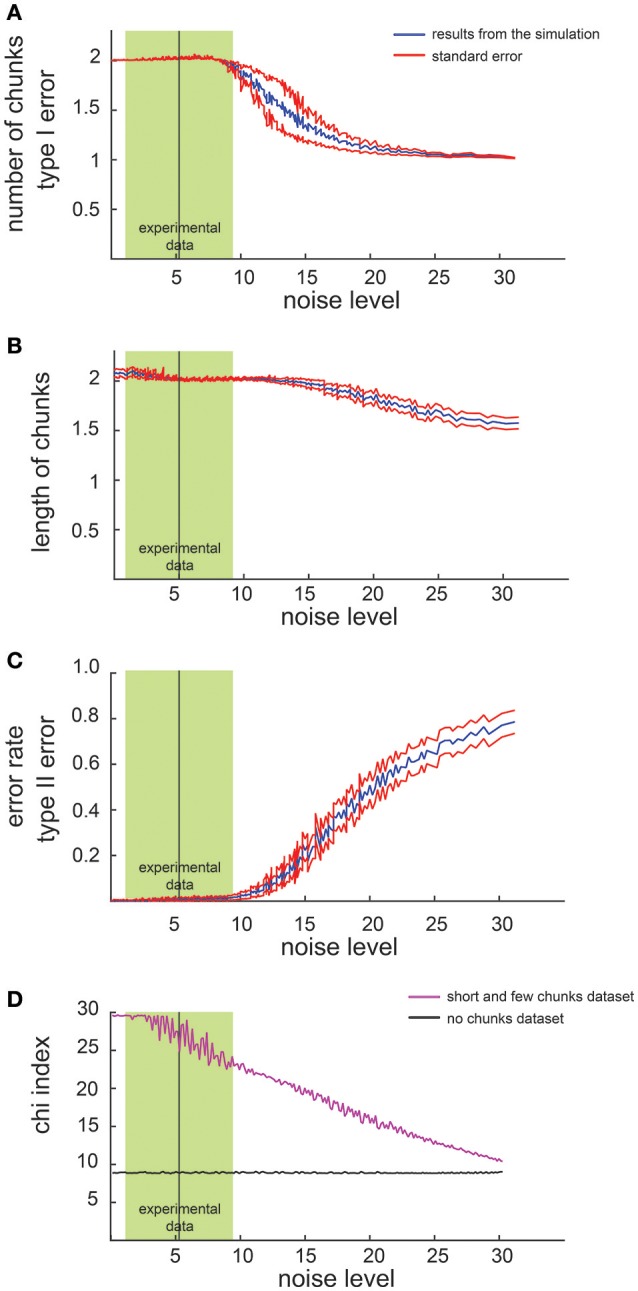
**Results of simulated dataset analysis**. Average number of chunks identified by the algorithm in the simulated dataset (few and short chunks). The x axis represents the noise level whereas the y axis represents the number of identified chunks **(A)** the chunk length **(B)**, the error rate **(C)** and the chi^2^-index **(D)**. The red lines indicate the standard error while the green shaded zone designates the range of noise level observed in dataset #1 (mean ± 2 SD).

We first examined the performance of the non-parametric chunking algorithm on 1000 iterations of simulated data, generated randomly for each combination of the noise parameters (i.e., the *slope* and the *intercept*). **Figure 3** reports the results for the simulated dataset with few and short chunks; similar results were obtained for the other datasets (few and long, many and long or many, and short chunks) and were therefore not illustrated. A complete representation of all the results is reported in supplementary materials (see Figures S2, S3). Figure 2 shows examples of results from the algorithm for 3 different noise levels in RT, whereas Figure 3 shows the global capacity of our algorithm to detect the number of chunks, their length and position, as a function of noise level. The noise level was computed from a large range of slope and intercept values (see Methods). When the noise remained confined to the range observed in actual experimental datasets (green shaded area in Figure 3), the estimated chunk number was unbiased (the average outcome of the 1000 iterations was equal to the real number of chunks). Similarly, regarding chunk lengths, our algorithm detected the correct lengths in a wide range of noise level, and particularly for noise parameters corresponding to the actual values measured in dataset #1.

Regarding the errors in determining the correct chunk position (Figure 3C), we averaged the sequence-wise error rate, i.e., the number of times the algorithm detected a non-existing chunk (type I error), or missed an actual chunk in a sequence (type II error). Within the range of noise parameters found in the experimental datasets, the average error rate was very close to zero, meaning that the algorithm committed few errors in all simulated conditions.

Furthermore, we compared the chi^2^-values computed on the dataset with no chunks and on the simulated data (Figure 3D): a significant difference (i.e., a lack of overlap of the confidence intervals), confirmed the reliability of the chi^2^-index in detecting a pattern in our simulated data.

Eventually, we investigated how the performance of the algorithm changes as a function of the number of sequences in each block. Not surprisingly, the results revealed that the higher the number of sequences per block, the better the results. Indicatively, a series of 4 sequences per block would be the minimum to have reliable results.

### Experimental dataset–comparison with previous methods

Different techniques have been used to analyze the data in the previous studies: in Solopchuk et al. (2016) correlations between RT series were used to estimate the chunking consistency across blocks, in Alamia et al. (2016) a clustering analysis based on network modularity was used to estimate the chunking dynamics during the experiment and finally in Clerget et al. (2012) chunks were identified by means of an ANOVA. Since the main advantage of the algorithm is that it provides both an estimation of the chunks positions in the sequence and an index that tracks the overall chunking strategy, we were able to compare our results with those obtained with these various analysis approaches.

**Figure 4:**
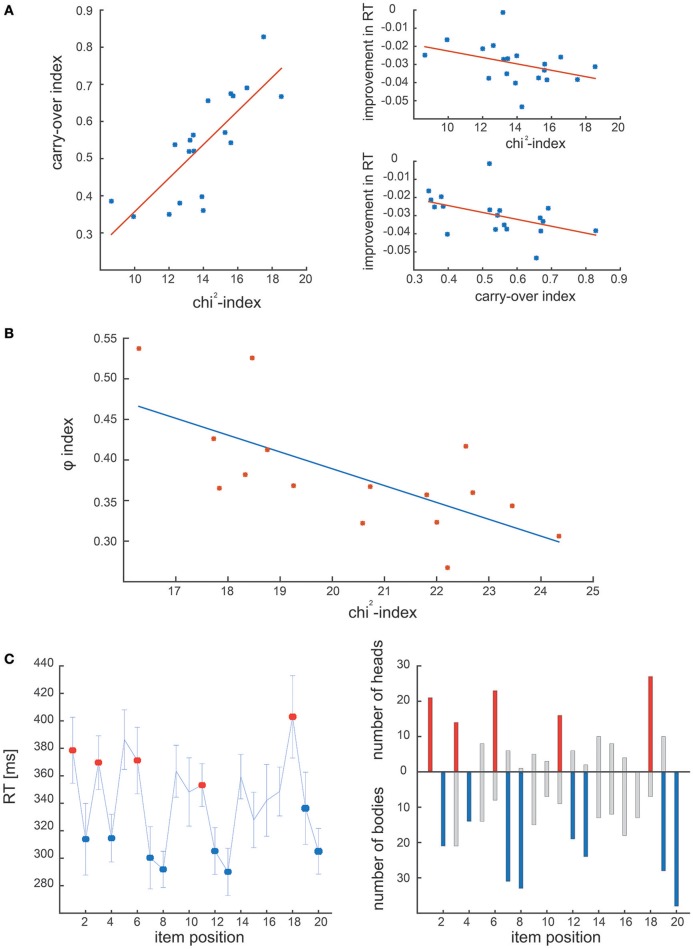
**Results of experimental dataset analysis. (A)** Correlation between the carry-over index and the chi^2^-index in the first experimental dataset. The two measures correlate, and are both correlated with the performance during the task (i.e., improvement in RT). **(B)** Correlation between φ-index and chi^2^-index obtained on the second experimental dataset. The two indexes are significantly correlated, confirming that both address the dynamics of the chunking strategy. **(C)** Comparison of the chunk positions in Clerget et al. (2012), identified by the ANOVA of RT series (Clerget et al, left panel) and the ones obtained with our algorithm (right panel). On both panels, chunk heads and bodies identified in Clerget et al., are denoted by red and blue colors, respectively.

Figure 4A shows the results of the comparative analysis of dataset #1: in the original study chunking strategy was quantified by means of the chunk carryover index, which represents the consistency of the chunking strategy across blocks (Solopchuk et al., 2016). We hypothesized that chunk carryover indexes would be related to the block-wise averaged chi^2^-indexes, as they both reflect chunking consistency. Indeed, we found them to be highly correlated (*r* = 0.8123, *p* < 0.001, Spearman). Furthermore, in the original study, chunk carryover was found to be correlated with performance improvement (quantified as decrease in the overall RT). In line with these results the chi^2^-index also correlated significantly with the improvements in the overall RT (*r* = −0.5316, *p* = 0.02, Spearman).

The second dataset was originally analyzed by means of a community detection analysis, in which individual RT patterns were organized in a network, characterized by the φ index, computed as the inverse of the network modularity. As reported in the first study that used this approach (Wymbs et al., 2012), higher values of φ suggest the absence (or concatenation) of chunks, whereas lower values of φ indicate the presence of several small chunks (or chunk segmentation), associated with higher RT variability. We predicted that the φ index would be highly correlated with our chi^2^-index, which tracks the consistency of the chunk strategy within each block, as the low values of chi^2^ indicate few and/or inconsistent chunks, whereas high values suggest many and/or consistent chunking patterns. Not surprisingly, the two indexes obtained from dataset #2 correlate significantly with each other (see Figure 4B; *r* = −0.6824, *p* < 0.01, Spearman).

Finally, Figure 4C shows the results of the comparative analysis of dataset #3 (Clerget et al.). The left panel illustrates the chunks identified by the Clerget approach, that is an ANOVA on the last block of RTs, whereas the right panel shows the chunks detected by our algorithm (represented as the number of times a given sequence item was identified as a chunk head or body). The comparison reveals that the general chunking pattern identified by these different approaches is the same. Moreover, our algorithm reveals considerable inter-subject variability of the chunking strategies. This ability to detect individual chunking patterns empowers an investigator to perform more sensitive data analysis, as it does not rely on a critical assumption of homogeneity of chunking strategies, thus, does not require large sample sizes for robust chunk detection.

The detailed statistical analysis of the measures provided by our algorithm (i.e., number and length of chunks, chi^2^-index) on the 3 experimental datasets is provided in supplementary materials.

## Discussion

The aim of this study was to develop a new method to identify patterns in RT series. In the first part of this study, we evaluated the performance of our algorithm by testing it on simulated datasets that consisted of 16-item sequences with known chunk features. The algorithm proved to be reliable to isolate chunks, particularly when tested with additive noise that remained within the range of magnitude observed in actual data. In the second part of this study, we tested our algorithm on three real datasets previously acquired in our lab (Clerget et al., 2012; Alamia et al., 2016; Solopchuk et al., 2016).

Despite a considerable literature on chunking, there is still no consensus on a gold standard method to analyze RT series in sequence learning; rather, several different methods have been used so far, often leading to results that are difficult to compare to each other. The different methods proposed in the literature can be classified into two main categories: those providing positions and number of chunks in each block, analyzing the data in a sequence-specific manner, and those providing, for each block, an index reflecting the “strength” of the chunk strategy used by the participants. Regarding the first group of methods, one approach consists in comparing, for each pair of consecutive items, the mean RT gathered for a given block, thus possibly identifying a significant difference between item RT that would be indicative of a chunk (Bo and Seidler, 2009; Clerget et al., 2011, 2012, 2013). However, this straightforward approach has a few drawbacks, mainly related to: (1) its sensitivity to outliers, (2) the small sample size (i.e., the number of sequences per block) used to perform the comparison between pair of items. The second approach, providing a global index of chunking across blocks, can be based on different methods. For example, Wymbs et al. (2012) used a method originally designed to identify communities in large networks, and which is based on the computation of a modularity index, defined as the degree to which a network can be divided into small clusters (Blondel et al., 2008; Mucha et al., 2010). In the context of sequence learning, this approach provides an index allowing us to determine how “modular” is a given sequence, or in other words, how easily the items of that sequence can be grouped into chunks. Recently, the same approach has been used to investigate the consequence of a disruption of Broca's area performed with transcranial magnetic stimulation on the chunking strategy (Alamia et al., 2016). The main advantage of this approach derives from the use of a single feature of the network (the index of modularity), which allows considering all the data at once, reducing considerably the contribution of the outliers. Nonetheless, this approach provides only an overall index that estimates the modularity of the RT series, regardless of the actual presence of chunks in the data; therefore this approach cannot be used to estimate chunk**s** length and number. In particular, the modularity index is affected not only by the number of chunks, but also by variations in the relative difference between items in RT: for example, the same chunking pattern can lead to different modularity index values just by having the first item RT vary, without unveiling an actual change in the chunking strategy.

Another method providing an index to assess the chunking strategy has been proposed by Tremblay et al. (2010), and is based on the variability of the RT data, estimated through the Eta Squared index. As for the previous method, this approach leads to a unique score reflecting the chunking strategy, without providing any further information about the size, position and number of chunks in the sequence. Another method based on a single index has been recently used in two studies (Song and Cohen, 2014; Solopchuk et al., 2016): this so-called “chunk carryover” index, provides a measure of the coherence of the chunking strategy across blocks, and is based on linear correlations between RT series: the stronger the correlation the more consistent the chunking pattern. Finally, a more complicated model has been proposed recently (Acuna et al., 2014) in which the RT series are modeled according to different factors, ranging from practice effect to biomechanical factors, and include the chunking structure. The advantage of this approach is that it combines information on the RT and on the error rate. However, in most sequence learning experiments, the training reduces considerably the error rate after few blocks, making its inclusion in the analyses less informative. Moreover, since this method relies on an optimization procedure, its downside is to require a large amount of data to grant optimal fits.

Crucially, the main novelty of our algorithm is to combine the advantages of the two aforementioned approaches. Indeed, it provides both individual chunk features, such as their position, length and number, and an overall index—the chi^2^-index—allowing to estimate the chunking “strength” across block repetitions. However, the main drawback of this algorithm, similarly to other methods used to analyze RT series, is its sensitivity to the number of sequences per block: if a block comprises less than 4 sequence repetitions, it is more likely to have false positive chunk detection, because of a higher sensitivity to outliers. Our findings suggest that 4 repetitions per block provide the right tradeoff between time resolution (number of sequences) and statistical reliability (false positive detection).

The results gathered by our new algorithm corroborate and extend the findings reported in earlier publications from our group (Clerget et al., 2012; Alamia et al., 2016; Solopchuk et al., 2016). In the work of Solopchuk et al. (dataset #1), chunking was shown to play a key role in improving sequence processing (Solopchuk et al., 2016), and we confirmed that conclusion by showing a correlation between the chi^2^-index and performance (Figure 4A). However, whereas the chunk carryover index, used as an estimate of chunking (Solopchuk et al., 2016) conveys information about the reliability of the chunking strategy during the whole experiment (being a mean correlation coefficient between block-wise averaged RT patterns), the chi^2^-index introduced in the present study provides a block by block evaluation of chunking, and thus a better time resolution to track chunking progression over block repetition. The aim of the second study from which we reanalyzed the data (Alamia et al., 2016) was to test the hypothesis that Brodmann area 44 (BA44) plays a central role in processing high-level chunking. To do so, Alamia et al. applied TMS over that cortical area in order to alter its excitability, and investigated how this manipulation affected sequence learning/processing; these results were compared to those of a control group that received TMS over a control area. The present study also supports the conclusion that BA44 is involved in chunk processing by showing a different chunking pattern in the two groups (Figure S1). Finally, the analysis of the last dataset showed no difference in the elementary chunking strategy between the two groups, but a significant difference in learning, highlighted through an interaction between BLOCK and GROUP on the number of chunks, confirming the findings of the original study (Clerget et al., 2012).

In addition to confirming our earlier findings, applying the novel algorithm we developed on the previously acquired datasets suggested two results of particular interest, namely, the existence of a relationship between chunking and performance, and a lack of decrease in chunk number across block repetitions. Recent studies have suggested that the accuracy in sequence performance and the decrease in RT are not affected by the reliance on chunking, suggesting a lack of functional benefit of chunking in sequence processing (Wymbs et al., 2012; Song and Cohen, 2014). In contrast, our analysis of datasets #1 and #2 provided strong evidence in favor of a positive correlation between performance (number of correct responses in the task) and the consistency of the chunking strategy (e.g., measured by means of the chi^2^-index) while learning the perceptual sequence. However, we failed to find a correlation in dataset #3, a finding which was thus in accordance with earlier results (Wymbs et al., 2012; Song and Cohen, 2014). This discrepancy could emerge from the nature of the tasks, since datasets #1 and #2 relied on an explicit symbolic sequence learning task, whereas dataset #3, similarly to earlier studies, involved motor sequences learned implicitly. Another important difference with the earlier studies was the length of the training underwent by the participants: in our dataset only 8 blocks of either 5 or 6 repetitions were considered, whereas in the studies of Wymbs et al and Song and Cohen, participants were usually trained to perform the sequence for several days. It is possible that the chunking process affects behavior mostly in the early stage of sequence learning, but not on the long run, when the sequence is over-learnt and when the performance has already reached a plateau.

The second interesting result that emerged from re-analyzing our previous experimental datasets concerns the lack of evidence in favor of any decrease in the number of chunks across block repetition, in contradiction with the view that concatenation of chunks should occur progressively during repeated execution of the sequences (Klapp, 1995; Verwey, 1996; Wymbs et al., 2012). In our experiments, and in line with the results of (Song and Cohen, 2014), no evidence emerged in favor of the concatenation hypothesis. Instead, the number of chunks and their length remained remarkably constant during the whole experiment, pointing rather toward a stability of the chunking strategy used by the participants. As suggested above, the discrepancy with earlier results (Wymbs et al., 2012) could be explained by the training duration. Another potential explanation for the difference in results could be the actual sequence that the participants had to learn: if the sequence length is not demanding (only 16 or 20 items, with a relatively easy sequence structure to memorize) the number of chunks could remain stable, and the initial chunking strategy could be already optimal in alleviating the working memory load, making the concatenation process not necessary.

In conclusion, we propose an original and reliable method to detect the position and length of chunks, and an index to track the chunking strategy of the participants during sequence learning tasks. Interestingly, analyzing three experimental datasets we reported a correlation between the chunking strategy and the task performance, and, notably, we found no evidence supporting chunk concatenation in the actual datasets we analyzed with this new algorithm.

## Author contributions

All authors listed, have made substantial, direct and intellectual contribution to the work, and approved it for publication.

## Funding

This work was performed at the Institute of Neuroscience (IoNS) of the Université catholique de Louvain (Brussels, Belgium); it was supported by grants from the ARC (Actions de Recherche Concertées, Communauté Française de Belgique), from the Fondation Médicale Reine Elisabeth (FMRE), and from the Fonds de la Recherche Scientifique (FNRS–FDP) to EO and AZ. AA is a Research Fellow at the FNRS and AZ is a Senior Research Associate supported by INNOVIRIS.

### Conflict of interest statement

The authors declare that the research was conducted in the absence of any commercial or financial relationships that could be construed as a potential conflict of interest.
