# Supplementary material for: Non-parametric Algorithm to Isolate Chunks in Response Sequences
Source: Front Behav Neurosci. 2016 Sep 21;10:177. doi: 10.3389/fnbeh.2016.00177 (PMC5030762; doi:10.3389/fnbeh.2016.00177)
Supplement: Supplementary file 1 [file DataSheet1.docx]

**Supplementary materials**

*Experimental dataset – analysis of the results*

We performed three GLMM considering either the number of chunks, their length or the **chi^2^** value as dependent variables on each of the 3 experimental datasets. The BLOCK factor was included as continuous independent variable, whereas GROUP was added as categorical factor when appropriate (i.e. in datasets # 2 and #3).

Regarding the **number of chunks** (Fig. 4)**,** in dataset #1 the results revealed an effect close to be significant for the BLOCK factor (F(1,24)=3.89, p=0.0603), while it was not significant for the two other datasets (dataset #2: F(1,8)=0.04, p=0.8533, dataset #3: F(1,7)=0.67, p=0.4394). Regarding the GROUP factor and the interaction, we did not observe any significant effect (dataset #2: GROUP F(1,8)=0.01, p=0.9236, interaction F(1,8)=0.20, p=0.6639; dataset #3: GROUP F(1,7)=1.59, p=0.2478, interaction F(1,7)=1.76, p=0.2259). To investigate further the effect of BLOCK in each dataset, we computed the Bayes Factor (BF) for the comparison of the models with and without the BLOCK factor. Such analysis confirmed the results of the GLMM: for all the datasets, the BF provided mild evidence in favor of the null hypothesis, i.e. a lack of effect of the factor BLOCK (dataset #1: BF=7.3891, p=0.1192, dataset #2: BF=49.4, p=0.0198, dataset #3: BF=11.0232, p=0.0832). Similarly, the BLOCK factor failed to affect **chunk length** (Fig. S1) (dataset #1: BLOCK F(1,24)=3.25, p=0.0839; dataset #2: BLOCK F(1,8)=1.76, p=0.2215, GROUP F(1,8)=0.54, p=0.4837, interaction F(1,8)=0.52, p=0.4894; dataset #3: BLOCK F(1,7)=3.68, p=0.0967, GROUP F(1,7)=0.18, p=0.6826, interaction F(1,7)=0.06, p=0.8183). The BF confirmed a lack of significance in the block effect for dataset #2 and #3 (dataset #2: BF=44.7012, p=0.0219; dataset #3: BF=24.5325, p=0.0392), but was not conclusive for dataset #1 (BF=0.5769, p=0.6341). In summary, the analyses performed with our algorithm showed an absence of BLOCK effect on both the length and number of chunks.

Regarding the **chi^2^**-values (Fig. 4), we performed the same GLMM with BLOCK and GROUP as factors, when appropriate. Concerning the first dataset, the GLMM showed a lack of BLOCK effect on the **chi^2^** value (BLOCK F(1,24)=2.06, p=0.1645), while in the second dataset, the BLOCK factor was significant (BLOCK F(1,8)=6.49, p=0.0344, GROUP F(1,8)=0.47, p=0.5122, interaction F(1,8)=0.24, p=0.6374). Finally, the same analysis performed on dataset #3 did not reveal any effect on the factor BLOCK (BLOCK F(1,7)=0.00, p=0.9700) but a significant interaction between the GROUP and BLOCK factors (GROUP F(1,7)=4.33, p=0.0760, interaction F(1,7)=8.38, p=0.0232). Finally, we correlated the **chi^2^** indexes with the performance computed as the percentage of correct responses per block, and with the average of the RT, block by block (Fig. 4). Interestingly, we found a significant correlation between the **chi^2^**-index and both the performance and RT in dataset #1 (performance: R=0.4522, p<0.0001; RT: R=-0.3520, p<0.0001) and in dataset #2 (performance: R=0.3363, p<0.0001, RT: R=-0.2084 p=0.01). No significant correlation was found in dataset #3, probably due to a difference between the tasks: symbolic sequence and explicit learning in dataset #1 and #2, and motor sequence and implicit learning in dataset #3. In conclusion, the analysis of the **chi^2^**-indexes confirms that chunking benefits overall task performance.

*Figure S1 – Theoretical method to estimate the threshold in the distribution of ranks difference*

A: The figure shows, step by step, the procedure to estimate the threshold in the ranks difference, when k=3 and n=16. A1. The uniform distribution (A1) is convolved k times (A2 and A3), then the result is convolved with itself and centered on zero in order to estimate the distribution of ranks difference (A4). The value of the cumulative distribution (A5) of this final distribution at α (set to 0.05 and displayed in red in the picture) returns the value of the threshold.

B: The figure shows, for different values of number of sequence repetitions per block k (x-axis) and number of items in the sequence N (different colors in the plot), the threshold obtained from the theoretical distribution (dashed lines) and the ones obtained from the rule of thumb formula (solid lines).

*Figure S2 - Experimental dataset results: number of chunks and correlation with performance*

A, B & C: Results from the three experimental datasets (from A to C), considering the number of chunks and the **chi^2^** values plotted block by block (first row), and the correlations between **chi^2^** values and performance, and **chi^2^** values and RT (second row). For datasets #2 and #3, the two groups are shown separately in different colors (blue for the control group, red for the experimental group).

*Figure S3 - Experimental dataset results: length of chunks and ranks*

Results of each experimental dataset: to the left - the length of chunks identified by the algorithm plotted against the block number, to the right - the average ranks for the whole population (dataset 1, panel A; dataset 2, panel B; dataset 3, panel C). In B and C the two groups are represented in two different colors (red for control group, blue for experimental group).
